# Deviant facial nerve course in the middle ear cavity^☆^

**DOI:** 10.1016/j.bjorl.2015.03.011

**Published:** 2015-09-07

**Authors:** Jungkyu Cho, Nayeon Choi, Sung Hwa Hong, Il Joon Moon

**Affiliations:** Department of Otorhinolaryngology-Head and Neck Surgery, Samsung Medical Center, Sungkyunkwan University School of Medicine, Seoul, Republic of Korea

## Introduction

Anomalous facial nerve (FN) course can be found in a significant number of cases with aural anomalies. The most common anomaly of the FN involves the tympanic portion overlying the oval window.^1^, ^2^, ^3^ Facial canal dehiscence of the tympanic portion may be responsible for the anomalous course of FN over the oval window. The incidence of facial canal dehiscence found during otologic surgery is relatively frequent and is usually related with cholesteatomas.^4^, ^5^ Aberrant FN course in a patient without accompanying anomaly or cholesteatoma has been demonstrated in a previous case report.^6^ However, the patient had not undergone imaging evaluation. Herein, the authors report an abnormal FN course in the tympanic portion, without any other associated anomalies.

## Case report

**Figure 1:**
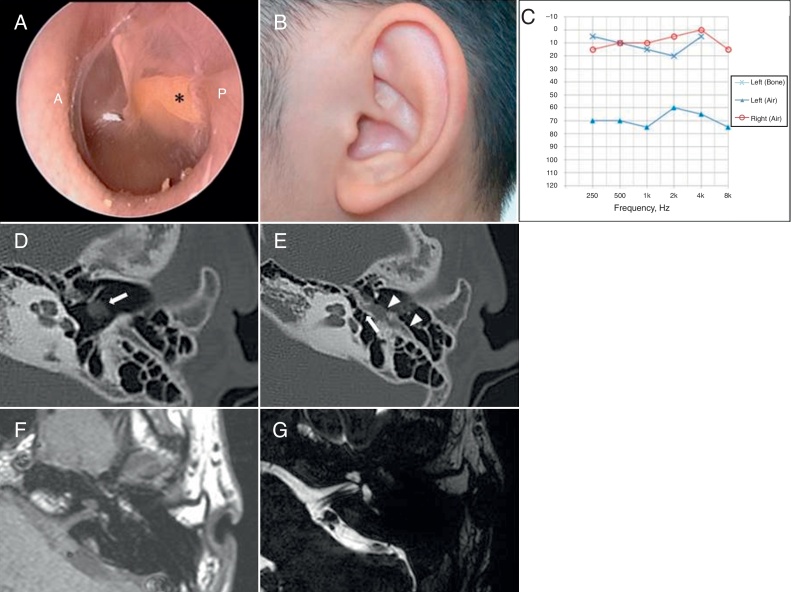
(A) Otoscopic examination reveals streak-like structure (asterisk) behind the eardrum. Yellowish bundle-like structure is placed at postero-superior quadrant. P, posterior canal wall; A, anterior canal wall. (B) External ear shows normal structure without anomaly. (C) Pure tone audiometry shows conductive hearing loss; air-bone conduction gap of 58 dB in the left ear. (D) Facial nerve dehiscence of tympanic segment, which is seen behind the tympanic membrane (white arrow). (E) Incudostapedial dislocation (white arrow) with lateralized facial nerve (white arrow head). (F) Intermediate signal intensity of the aberrant facial nerve course. (G) Well-delineated facial and vestibulocochlear nerve in internal auditory canal.

An 18-year-old male presented to the outpatient clinic with left-sided non-progressive hearing loss since childhood. Otoscopic examination revealed bundle-like structure behind the posterior portion of the tympanic membrane (Fig. 1A). Other physical exams showed no facial nerve palsy or auricular anomaly (Fig. 1B). Pure-tone average (0.5, 1, 2, and 3 kHz) showed air-bone gap of 58 dB (average bone-conduction threshold = 3.75 dB, average air-conduction threshold = 62.5 dB) (Fig. 1C). Accordingly, computed tomography (CT) scan of the temporal bone was performed for evaluation of the middle ear cavity along with ossicular structures. CT revealed a hypoplastic middle ear cavity, incudostapedial joint separation, and lateralization of the tympanic segment of the facial nerve, which was observed behind the tympanic membrane (Fig. 1D and E). Internal auditory canal MRI demonstrated no anomalies in the inner ear (Fig. 1F and G). Since aberrant course of the tympanic segment of facial nerve was identified, further surgical exploration was deferred.

## Discussion

Most patients with FN anomaly do not have any clinical symptoms. Conductive hearing loss, mainly due to associated ossicular disruption, may be the only clinical presentation. Therefore, it is difficult to suspect middle ear mass as an unusual presentation of the FN course or even as a structural or passage anomaly, especially when other associated anomalies are not noted. Furthermore, cases of FN dehiscence shown in previous studies were mainly associated with cholesteatomas or congenital aural atresias.^2^, ^3^, ^5^

However, aberrant course of FN lateral to the ossicles without accompanying auricular anomaly was reported in a prior case.^6^ Hence, it is advisable not to exclude aberrant FN pathway in patients without accompanying anomaly or other middle ear disease. In the previous report of aberrant FN course without other anomalies, imaging evaluation was not performed preoperatively. Surgical exploration with electrical stimulation monitoring was used to confirm the middle ear mass as the FN.^6^ However, these surgical procedures in the middle ear cavity could lead to FN damage. Thus, if anomalous FN is suspected, preoperative imaging evaluation is mandatory. In the present case, radiologic evaluation was performed before planning surgical exploration.

CT imaging provides precise prediction of the FN course, coinciding with surgical findings in most cases of congenital aural atresia.^2^ Therefore, the primary imaging modality for evaluation of the FN course should include high resolution CT. In previous studies of anomalous FN of the tympanic portion, the FN runs more anterolaterally in the middle ear cavity than in normal patients.

Therefore, physicians should suspect the mass located in the posterior middle ear cavity as a variation of the FN pathway and perform imaging evaluation before planning interventions.^1^, ^2^, ^4^, ^6^

## Final comments

Unusual structure seen through the tympanic membrane should be evaluated by imaging before definitive treatment. CT is especially useful for this purpose, because the tympanic segment of the FN lying lateral to the ossicles can be easily identified. If aberrant course of the FN is suspected by symptoms and clinical findings, physicians should be cautious in determining surgical interventions, such as ventilation tube insertion or explorative tympanotomy.

## Conflicts of interest

The authors declare no conflicts of interest.

## References

[1] Fu Y., Zhang T. (2011). Facial nerve lying lateral to ossicles in one case of congenital aural atresia. Int J Pediatr Otorhinolaryngol.

[2] Yu Z., Han D., Gong S., Wang Z., Dai H., Zhao S. (2008). Facial nerve course in congenital aural atresia – identified by preoperative CT scanning and surgical findings. Acta Otolaryngol.

[3] Huang B.R., Juan C.J., Wang C.H. (2008). Infantile facial nerve course in an adult patient with congenital aural dysplasia. Otolaryngol Head Neck Surg.

[4] Selesnick S.H., Lynn-Macrae A.G. (2001). The incidence of facial nerve dehiscence at surgery for cholesteatoma. Otol Neurotol.

[5] Di Martino E., Sellhaus B., Haensel J., Schlegel J.G., Westhofen M., Prescher A. (2005). Fallopian canal dehiscences: a survey of clinical and anatomical findings. Eur Arch Otorhinolaryngol.

[6] Kuo C.Y., Wang C.H. (2012). Aberrant facial nerve exposed behind the eardrum. Otolaryngol Head Neck Surg.

